# Factors influencing dietary behaviours in urban food environments in Africa: a systematic mapping review

**DOI:** 10.1017/S1368980019005305

**Published:** 2020-10

**Authors:** Hibbah Osei-Kwasi, Aarti Mohindra, Andrew Booth, Amos Laar, Milka Wanjohi, Fiona Graham, Rebecca Pradeilles, Emmanuel Cohen, Michelle Holdsworth

**Affiliations:** 1 Public Health Section, School of Health and Related Research (ScHARR), University of Sheffield, Sheffield S14DA, UK; 2 Department of Clinical Sciences and Nutrition, University of Chester, Chester, UK; 3 Department of Population, Family & Reproductive Health, School of Public Health, University of Ghana, Accra, Ghana; 4 African Population and Health Research Center, Nairobi, Kenya; 5 South African Medical Research Council/Wits Developmental Pathways for Health Research Unit, Department of Paediatrics, Faculty of Health Sciences, University of the Witwatersrand, Johannesburg, South Africa; 6 Unité Mixte Internationale 3189 Environnement, Santé, Sociétés, Faculté de Médecine-secteur Nord, Centre National de la Recherche Scientifique, Marseille, France; 7 Joint Research Unit on Food & Nutrition Research Global South, French National Research Institute for Sustainable Development (IRD), Montpellier, France

**Keywords:** Dietary behaviour, Africa, Urban, Food environment

## Abstract

**Objective:**

To identify factors influencing dietary behaviours in urban food environments in Africa and identify areas for future research.

**Design:**

We systematically reviewed published/grey literature (protocol CRD4201706893). Findings were compiled into a map using a socio-ecological model on four environmental levels: individual, social, physical and macro.

**Setting:**

Urban food environments in Africa.

**Participants:**

Studies involving adolescents and adults (11–70 years, male/female).

**Results:**

Thirty-nine studies were included (six adolescent, fifteen adolescent/adult combined and eighteen adult). Quantitative methods were most common (twenty-eight quantitative, nine qualitative and two mixed methods). Studies were from fifteen African countries. Seventy-seven factors influencing dietary behaviours were identified, with two-thirds at the individual level (45/77). Factors in the social (11/77), physical (12/77) and macro (9/77) environments were investigated less. Individual-level factors that specifically emerged for adolescents included self-esteem, body satisfaction, dieting, spoken language, school attendance, gender, body composition, pubertal development, BMI and fat mass. Studies involving adolescents investigated social environment-level factors more, for example, sharing food with friends. The physical food environment was more commonly explored in adults, for example, convenience/availability of food. Macro-level factors associated with dietary behaviours were food/drink advertising, religion and food prices. Factors associated with dietary behaviour were broadly similar for men and women.

**Conclusions:**

The dominance of studies exploring individual-level factors suggests a need for research to explore how social, physical and macro-level environments drive dietary behaviours of adolescents and adults in urban Africa. More studies are needed for adolescents and men, and studies widening the geographical scope to encompass all African countries.

Rapid demographic change in Africa, partly driven by increasing migration of individuals into cities, has changed people’s food environments and dietary habits^(1)^. Economic development has increased access to food markets selling energy-dense processed foods at low prices and decreased the price of certain foods such as vegetable oils^(2)^. Modification of diet structure towards a higher intake of energy-dense foods (especially from fat and added sugars), a higher consumption of processed foods^(3)^, animal source foods, sugar and saturated fats, and a lower intake of complex carbohydrates, dietary fibre, fruit and vegetables has led to a significant change in diet quality over the past 20 years^(4)^. The nutrition transition in urban areas of many African countries has resulted in a ‘double burden of disease’ in which there is an increased prevalence of nutrition-related non-communicable diseases (NR-NCD) alongside existing communicable diseases. Although obesity prevalence is higher among African women than men, there has been a rise in both^(5,6)^. Children and adolescents are an important group to target in the prevention of overweight and obesity^(7)^. In 2010, of the 43 million children estimated to be overweight and obesity, 35 million were from low- and middle-income countries^(7)^. The prevalence of overweight and obesity in children in Africa is expected to increase from 8·5 % (2010) to a projected 12·7 % by 2020. By understanding this shift in nutrition and disease, new NR-NCD prevention strategies that account for the factors driving dietary behaviours can be developed across the life course.

A mapping review was previously conducted in 2015^(8)^ to identify drivers of dietary behaviours specifically in adult women within urban settings in African countries and identify priorities for future research. However, the increasing evidence that the overweight and obesity burden is spread more widely across population groups indicates the need for a broader review. Hence, this systematic review mapped the factors influencing dietary behaviours of adolescents and adults of both genders in African urban food environments and identified areas for future research.

## Methods

A systematic mapping review^(9)^ was conducted to map existing literature regarding factors influencing dietary behaviours in urban Africa. Systematic mapping reviews are often conducted as a prelude to further research and are imperative in the identification of research gaps. Prior to conducting the review, the Cochrane Database of Systematic Reviews and MEDLINE were searched to ensure that no similar reviews were underway or had been conducted beyond the original mapping review^(8)^. A review protocol was produced to ensure transparency in the review methodology and then registered with the PROSPERO database of existing and on-going systematic reviews (registration number CRD4201706893).

To determine appropriate inclusion and exclusion criteria for the review, the Sample, Phenomenon of Interest, Design, Evaluation, Research type tool was used^(10)^. Criteria used in the original review were modified to acknowledge the additional population groups (adolescents and adult men)^(8)^; otherwise, the same processes were applied to ensure compatibility.

### Inclusion and exclusion criteria

The original review conducted in 2015 investigated women aged 18–70 years living in urban Africa from 1971 to April 2015^(8)^. This current review synthesised recent research in this same group, published since April 2015 to April 2019, and included men (18–70 years) and female/male adolescents (11–17 years), between 1971 and April 2019. All participants were living in urban Africa, those from rural settings were excluded, as were studies with participants <11 years or >70 years. Participants with a clinical diagnosis related to NR-NCD were excluded; excluding studies with specific diseases also ensured that the included studies were of healthy African populations and not specific clinical sub-groups. The phenomenon of interest was defined as factors influencing dietary behaviours. This was purposely broad to enable sensitive mapping of all available literature. Furthermore, studies including African-Americans or African migrants to non-African countries were excluded on the basis of setting. Studies measuring the effect of factors on dietary behaviours were included, but studies that focused on the relationship between diet and diet-related diseases were excluded given the focus on factors influencing dietary behaviour rather than their effect on specific diseases.

To ensure broad coverage of research, all types of study designs were included, that is, randomised controlled trials, cohort studies, case–control studies, ecological/observational studies, reviews and meta-analyses. All publication types were included, provided they were in English or French. Languages were chosen to acknowledge the main publishing languages in Africa.

For adult men and adolescents, any appropriate study from 1971 to 2019 was included. For adult women, studies published since the previous search (April 2015–April 2019) were retrieved. The chosen 1971 start date reflected the earliest appearance of relevant publications concerning health behaviour in the context of the epidemiological transition^(11)^ on the nominated databases and search engines. The primary outcome was dietary behaviour, including macronutrient, food item and food diversity intake, as well as eating habits, preferences, choices and feeding-related mannerisms. Macronutrients were included because of the review’s focus on urban settings where dietary transition is more likely to be associated with dietary change from the nutrition transition, which is associated with increased consumption of fat, vegetable and edible fat and increased added sugar^(6)^.

### Search strategy

Electronic searches were conducted across six key databases: EMBASE, MEDLINE, CINAHL, PsycINFO, ASSIA and African Index Medicus. The search strategy replicated that used in the previous review with the additional inclusion of search terms representing adult men and adolescents^(8)^. An example of a search strategy used for these databases can be found in Supplemental Table 1 in the online supplementary material. Grey literature was explored through the WHO International Trials Registry Index and Thesis (UK and Ireland) Database.

Reference lists for the seventeen studies included in the initial review were examined, and citation tracking using Google Scholar (through Publish or Perish™) was also conducted. Forward and backward citation tracking sought to ensure that no important studies were missed and that representation of appropriate literature was maximised. Reference lists of newly identified included studies, reflecting the expansion of date range and populations of interest, were also reviewed. The dual approach of subject searching and follow-up citation tracking was considered to provide sufficient coverage of the relevant literature^(12)^.

### Study selection

Studies that fulfilled the inclusion and exclusion criteria for title and abstract then underwent full-text screening by two reviewers (A.M./F.G.). Duplicates were removed prior to full-text screening. A second reviewer (H.O.-K./M.H.) assessed 10 % of excluded studies at two stages: the title and abstract stage and the full-text search stage. Any disagreements were resolved by discussion. If no agreement was reached, a third reviewer also assessed the study.

### Quality assessment

Quality assessment is not a mandatory requirement for a mapping review^(9)^. However, by incorporating it into the review methodology, it enhances the credibility of the review’s findings and is particularly useful in documenting uncertainties that persist in relation to previous research^(9)^. Quality assessment was conducted with a validated tool^(13)^ for qualitative and quantitative studies by two reviewers independently (A.M., M.W. or F.G.).

### Data extraction

Data were extracted from included studies by one of two principal reviewers (A.M. or F.G.) supported by a second reviewer (H.O.-K. or M.H.) and was checked by a member of the review team (M.W.). As the aim of this mapping review was to map the factors influencing dietary behaviours of adolescents and adults living in African urban food environments and identify areas for future research, it was decided to include all factors reported by authors and not to restrict the review to reporting factors only where a statistical relationship or association had been demonstrated.

### Data synthesis

There are different approaches to updating a review. In this review, the new findings were integrated with those of the original review at the synthesis level^(14)^ in order to present all the evidence for men, women and adolescents for the same timescale. In order to determine which factors influence dietary behaviours in the three population sub-groups, factors influencing dietary behaviours for adults and adolescents of all thirty-eight studies were mapped to the socio-ecological model defined by Story *et al.* ^(15)^. Factors were placed within four broad levels: individual, social environment, physical environment and macro-environment and assigned to an appropriate sub-level. For novel factors that emerged, it was decided within the team where to place it in the aforementioned socio-ecological model, similar to the original review^(8)^. Reporting of the review followed the Preferred Reporting Items for Systematic Reviews and Meta-Analyses) checklist^(16)^.

## Results

### Search results

**Fig. 1:**
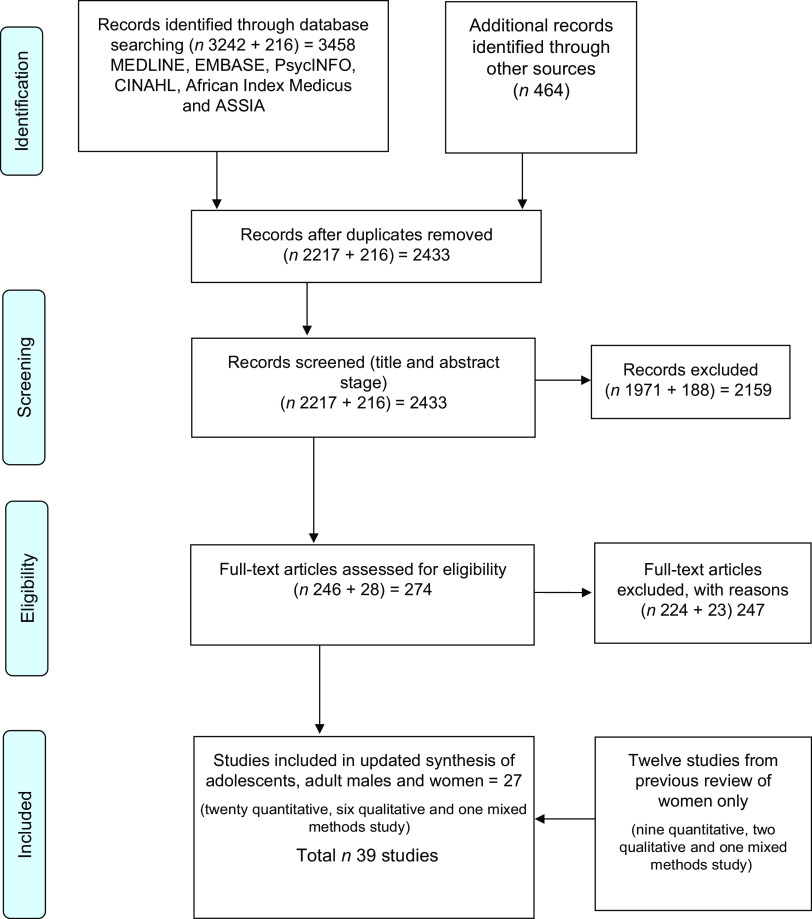
Preferred Reporting Items for Systematic Reviews and Meta-Analyses flow diagram showing the selection of studies for the present systematic mapping review

The search yielded 2433 title and abstract records after duplicates were removed (Fig. 1); 274 records remained for full-text retrieval, at which stage 247 records were excluded, leaving twenty-seven studies for inclusion for studies of adolescents, men and women (from 2015). Twelve studies from an earlier review of women only aged 18–70 years (1971–2015) were integrated in the review findings, giving a total of thirty-nine studies.

### Description of included studies

Thirty-nine studies were included in the final data synthesis (Table 1), of which nineteen were conducted in lower middle-income countries^(17)^: Cape Verde, Egypt, Ghana, Kenya, Morocco, Nigeria and Tunisia. Thirteen studies were conducted in upper middle-income countries: Botswana, Mauritius and South Africa, and one study was undertaken in the Seychelles (high-income country). Only six studies were undertaken in low-income countries: Burkina Faso, Benin, Niger and Tanzania (Table 1). Over half of studies were conducted in Ghana and Morocco (six studies each) or South Africa (ten studies).

**Table 1:** Characteristics of the included studies (39 studies and 45 records)

Study	Design, method	Country	Income level	Sample characteristics		Sample size	Sampling
Qualitative studies				Gender	Age (range)	*n*/households	
Batnitzky^(18)^	Field study, semi-structured interviews, observation	Morocco	Lower middle	Mixed	20+ years (adult)	1789	Unclear – individuals then households
Boatemaa *et al.* ^(19)^	Cross-sectional, interviews	Ghana	Lower middle	Mixed	15–35 years and 35+ years (adolescent and adult)	30	Purposive sampling
Brown *et al.* ^(20)^	Cross-sectional, focus groups	Botswana	Upper middle	Mixed	12–18 years (adolescent) and adult (age range not specified)	72–132 (adolescents) parents unknown	Sampling of schools with differing tuition status
Craveiro *et al.* ^(21)^	Observational, focus groups	Cape Verde	Lower middle	Mixed	18–41 years (adult)	48	Opportunistic sampling using probabilistic sampling with random selection
Draper *et al.* ^(22)^	Observational, focus groups	South Africa	Upper middle	Female	24–51 years (adult)	21	Convenience sampling
Legwegoh^(23)^ and Legwegoh & Hovorka^(24)^	Case-study, interview	Botswana	Upper middle	Mixed	20–65 years (adult)	40 households	Purposive sample, stratified based on household-head gender and socio-economic status
Rguibi & Behalsen^(25)^	Cross-sectional, questionnaire via interview	Morocco	Lower middle	Female	15–70 years (adolescent and adult)	249	Convenience. Women visiting primary care centres
Sedibe *et al.* ^(26)^ and Voorend *et al.* ^(27)^	Observational, duo-interviews	South Africa	Upper middle	Female	15–21 years (adolescent)	58	Voluntary participation following researcher involvement in school
Quantitative studies							
Agbozo *et al.* ^(28)^	Cross-sectional, questionnaire	Ghana	Lower middle	Mixed	60–70 years (adult)	120	Purposive sample from four peri-urban communities
Amenyah & Michels^((29)^	Cross-sectional, questionnaire	Ghana	Lower middle	Mixed	11–18 years (adolescent)	370	Random selection, five secondary schools
Aounallah-Skhiri *et al.* ^(30)^	Cross-sectional, questionnaire	Tunisia	Lower middle	Mixed	15–19 years (adolescent and adult)	1019	Clustered random sampling from three regions of Tunisia
Becquey *et al.* ^(31)^	Cross-sectional, questionnaire	Burkina Faso	Low	Mixed	15–65 years (adolescent and adult)	1072	Purposive random sampling
Cisse-Egbuonye *et al.* ^(32)^	Quantitative, cross-sectional	Niger	Low income	Female	15–49 years (adolescent and adult)	3360	Randomly selected household heads in purposive sample
Codjoe *et al.* ^(33)^	Cross-sectional	Ghana	Lower middle income	Mixed	15–59 years (adolescent and adult males), 15–49 years (adolescent and adult)	452 households	Purposive sampling according to age from a larger data set
El Ansari & Berg-Beckhoff^(34)^	Cross-sectional, questionnaire	Egypt	Lower middle	Mixed	16–30 years (adolescent and adults)	2810	Voluntary questionnaire distributed to students attending lectures of randomly selected courses
Feeley *et al.* ^(35)^	Cohort, questionnaire	South Africa	Upper middle	Mixed	13–17 years (adolescent)	1298	Cohort selection sampling-recruitment of all singleton births that occurred over a 7-week period in public delivery centres from all population groups
Fokeena & Jeewon^(36)^	Cross-sectional, self-reported questionnaires	Mauritius	Upper middle	Mixed	12–15 years (adolescent)	200	Multistage sampling, schools randomly selected from four educational zones of Mauritius and sample taken from three of these schools
Glozah & Pevalin^(37)^	Cross-sectional, self-reported questionnaires	Ghana	Lower middle income	Mixed	14–21 years (adolescent and adult)	770	Participants selected at random from four senior high schools that were purposively selected in Accra
Gitau *et al.* ^(38)^	Longitudinal, self-reported questionnaire	South Africa	Upper middle	Males	13–17 years (adolescent)	391	Stratified convenience sample
Hattingh *et al.* ^(39,40,41)^	Cross-sectional, questionnaire	South Africa	Upper middle	Female	25–44 years (adult)	488	Stratified random according to number of plots in each settlement
Jafri *et al.* ^(42)^	Cross-sectional, questionnaire	Morocco	Lower middle	Female	18+ years (adult)	401	Multistage cluster. Households randomly selected within clusters
Kiboi *et al.* ^(43)^	Cross-sectional, structured interviews, questionnaire	Kenya	Lower middle	Female	16–49 years (adolescent and adult)	254	Purposive sampling at antenatal clinic in a hospital over 1 month
Landais^(44)^ and Landais *et al.* ^(45)^	Cross-sectional, questionnaire	Morocco	Lower middle	Female	20–49 years (adult)	894	Multistage cluster. Households then addresses randomly selected from enumeration areas
López *et al.* ^(46)^	Observational, 3 × 24 h dietary recalls	Morocco	Lower middle	Mixed	15–20 years (adolescent and adult)	327	All students enrolled in high schools year 2007–2008 completed survey
Mayén *et al.* ^(47)^	Cross-sectional, survey	Seychelles	High	Mixed	25–64 years (adult)	2004	National surveys, random sample drawn from entire population
Mbochi *et al.* ^(48)^	Cross-sectional, questionnaire	Kenya	Lower middle	Female	25–54 years (adult)	365	Stratified random according to number of women in each socio-economic stratum
Mogre *et al.* ^(49)^	Cross-sectional, questionnaire	Ghana	Lower middle	Mixed	20–60 years (adult)	235	Stratified random based on number of employees in each department
Njelekela *et al.* ^(50)^	Cross-sectional, questionnaire	Tanzania	Low	Mixed	45–66 years (adult)	209	Random stratified selection from list of adult residents, strata: gender
Onyiriuka *et al.* ^(51)^	Cross-sectional, structured questionnaire	Nigeria	Lower middle	Female	12–19 years (adolescent and adult)	2097	Random selection by ballot from four all-girls schools, no sampling performed as designed to include all students
Peltzer & Phaswana-Mafuya^(52)^	Cross-sectional, survey	South Africa	Upper middle	Mixed	>50 years (adult)	3840	National population based sample, from original study (SAGE; two-stage probability sample)
Savy *et al.* ^(53)^	Cross-sectional, questionnaire	Burkina Faso	Low	Female	29–50 years (adult)	481	Random, from a database containing an exhaustive list of inhabitants
Sodjinou *et al.* ^(54,55)^	Cross-sectional, questionnaire	Benin	Low	Mixed	25–60 years (adult)	200	Multistage cluster. Neighbourhoods, households, then individuals randomly selected
Soualem *et al.* ^(56)^	Cross-sectional, questionnaires	Morocco	Lower middle	Mixed	12–16 years (adolescent)	190	Random selection from five schools in Gharb region
Steyn *et al.* ^(57)^	Cross-sectional, structured interview	South Africa	Upper middle	Mixed	≥16 years (adolescent and adult)	3287	Stratified sampling of annual survey data
Van Zyl *et al.* ^(58)^	Cross-sectional, questionnaire	South Africa	Upper middle	Mixed	19–30 years (adult)	341	Convenience, residents of Johannesburg visiting a mall
Waswa^(59)^	Cross-sectional, questionnaire	Kenya	Lower middle	Female	20–25 years (adult)	260	Stratified random according to university department size including each year
Zeba *et al.* ^(60)^	Cross-sectional, questionnaires	Burkina Faso	Low	Mixed	25–60 years (adult)	110	Stratified random sampling, stratification by income
Mixed-methods							
Charlton *et al.* ^(61)^	Cross-sectional, questionnaire; focus groups	South Africa	Upper middle	Female	Questionnaire: 17–50 years (adult and adolescent); Focus groups: 18–49 years (adult and adolescent)	Questionnaire: 394; focus groups: 39	Convenience, according to age and gender
Pradeilles^(62)^	Cross-sectional, questionnaires; focus groups	South Africa	Upper middle	Mixed	Questionnaire: 17–19 years (adult and adolescent); Focus groups: 18 years+ (adult)	Questionnaire: 631; focus groups: 51	Cohort selection sampling-recruitment of all singleton births that occurred over a 7-week period in public delivery centres from all population groups; Snowball sampling

Of the thirty-nine studies, eight were qualitative (ten records)^(18–27)^, twenty-nine (thirty-three records) were quantitative^(28–60)^ and two used mixed methods^(61,62)^ studies. The qualitative and quantitative data in the latter were extracted separately in order to generate distinct quality assessment scores. Of the thirty-nine studies, thirty-two were cross-sectional studies^(18–20,25,28–37,39–45,47–62)^, four were observational^(18,21,26,27,46)^, two used a longitudinal design^(38)^ and one was a detailed case study^(23,24)^. The methodology consisted of interviews and focus groups to obtain qualitative data, whereas self-administered or interviewer-led surveys were mostly used for quantitative studies.

### Quality assessment

In summary, while most of the quantitative studies scored high on criteria such as appropriate study designs, question/objective sufficiently described and data analysis clearly described, these studies did not report on controlling for confounders or estimation of variance in the main results.

Similarly, in all qualitative studies, authors failed to report on procedures to establish credibility or show reflexivity. The individual aspects of the quality assessment conducted for all thirty-nine included studies (see online supplementary material, Supplemental Tables 2 and 3).

#### Factors influencing diet or dietary behaviour in urban Africa

In total, seventy-seven factors influencing dietary behaviours were identified, with two-thirds at the individual level (45/77). Factors in the social (11/77), physical (12/77) and macro (9/77) environments were investigated less. Slightly more studies investigating social-level factors studied adolescent populations (Table 2). The configuration of dietary factors in adult men paralleled that of adult women, probably because relevant included studies examined a mixed adult population. In all population groups, the individual and household factors level of the socio-ecological model was the most studied.

**Table 2:** Factors in urban African food environments influencing dietary behaviours in the included studies (*n* 39)

Level	Sub-level	Factor (no. of studies)	Dietary behaviour	Evidence	Population
Individual and household (45)	Cognitions (12)	Taste (4)	Dietary intake	Pradeilles^(62)MM^, Sedibe *et al.* ^(26)QL^ and Voorend *et al.* ^(27)QL^	Mixed adolescent adult; Female adolescent
			Fast-food intake	Van Zyl *et al.* ^(58)QN^ *	Mixed adult
			Food choice	Charlton *et al.* ^(61)MM^	Female adolescent and adult
		Preferences (1)	Food choice	Boatemma *et al.* ^(19)QL^	Mixed adolescent and adult; female adolescent
		Hunger/not hungry/lack of appetite (6)	Fruit and vegetable intake	Peltzer & Phaswana-Mafuya^(52)QN^ *	Mixed adult
			Food intake	Agbozo *et al.* ^(28)QN^ *, Mogre *et al.* ^(49)QN^ * and Waswa^(59)QN^ *	Mixed adult; Mixed adult; Female adult
			Dietary diversity	Cisse-Egbuonye *et al.* ^(32)QN^ †	Female adolescent and adult
			Skipping meals	Onyiriuka *et al.* ^(51)QN^ †	Female adolescent
		Mood (1)	Food intake	Waswa^(59)QN^ *	Female adult
		Subjective health status (4)	Fruit and vegetable intake	Peltzer & Phaswana-Mafuya^(52)QN^ ‡ and Mogre *et al.* ^(49)QN^ *	Mixed adult; Mixed adult
			Food choice	Agbozo *et al.* ^(28)QN^ *	Mixed adult
			Dietary intake/Disordered eating	Amenyah & Michels^(29)QN^ *	Mixed adolescent
		Perceived stress (1)	Dietary intake	El Ansari & Berg-Beckhoff^(34)QN^ †	Mixed adolescent and adult
		Self-esteem (1)	Disordered eating	Gitau *et al.* ^(38)QN^ ‡	Males adolescent
		Body satisfaction (1)	Disordered eating	Gitau *et al.* ^(38)QN^ ‡	Males adolescent
		Body image perception (1)	Food intake	Waswa^(59)QN^ *	Female adult
		Food knowledge (3)	Fruit and vegetable intake	Landais^(44)^/Landais *et al.* ^(45)QN^ †	Mixed adult
			Food choice	Agbozo *et al.* ^(28)QN^ ‡	Mixed adult
			Food intake	Waswa^(59)QN^ *	Female adult
		Perception of diet quality (1)	Dietary diversity	Becquey *et al.* ^(31)QN^ †	Mixed adolescent and adult
		Perception of diet quantity (1)	Dietary diversity	Becquey *et al.* ^(31)QN^ †	Mixed adolescent and adult
	Lifestyle/behaviours (15)	Dieting (1)	Dietary habits	Sedibe *et al.* ^(26)^/Voorend *et al.* ^(27)QL^	Female adolescent
		Skipping meals (1)	Fruit and vegetable intake	Landais^(44)^/Landais *et al.* ^(45)QN^ *	Female adult
		Snacking (1)	Dietary diversity	Becquey *et al.* ^(31)QN^ †	Mixed adolescent and adult
		Habit/routine (1)	Food choice	Charlton *et al.* ^(61)MM^	Female adolescent and adult
		Household dietary diversity (1)	Dietary diversity	Cisse-Egbuonye *et al.* ^(32)QN^ †	Female adolescent and adult
		Processed food consumption (1)	Fruit and vegetable intake	Landais^(44)^/Landais *et al.* ^(45)QN^ *	Female adult
		Eating out occasions (1)	Fruit and vegetable intake	Landais^(44)^/Landais *et al.* ^(45)QN^ *	Female adult
		Eating three daily meals (1)	Fruit and vegetable intake	Landais^(44)^/Landais *et al.* ^(45)QN^ *	Female adult
		Overall lifestyle (1)	Diet quality	Sodjinou *et al.* ^(54)^/Sodjinou *et al.* ^(55)QN^ †	Mixed adult
		Spoken language (1)	Food quality	Soualem *et al.* ^(56)QN^ †	Mixed Adolescent
		Time limitations (5)	Dietary intake	Legwegoh^(23)^/Legwegoh & Hovorka^(24)QN^	Mixed adult
			Fast-food intake	Van Zyl *et al.* ^(58)QN^ *	Mixed adult
			Food choice	Brown *et al.* ^(20)QL^	Mixed adolescent and adult
			Unhealthy food intake	Craveiro *et al.* ^(21)QL^	Mixed adult
			Skipping meal	Mogre *et al.* ^(49)QN^ *	Mixed adult
		Quality of life (1)	Fruit and vegetable intake	Peltzer & Phaswana-Mafuya^(52)QN^ ‡	Mixed adult
		Tobacco use (2)	Fruit and vegetable intake	Peltzer & Phaswana-Mafuya^(52)QN^ †	Mixed adult
			Diet quality	Sodjinou *et al.* ^(54)^/Sodjinou *et al.* ^(55)QN^ †	Mixed adult
		Alcohol use (2)	Fruit and vegetable intake	Peltzer & Phaswana-Mafuya^(52)QN^ *	Mixed adult
			Diet quality	Sodjinou *et al.* ^(54)^/Sodjinou *et al.* ^(55)QN^ †	Mixed adult
		Physical activity (5)	Fruit and vegetable intake	Peltzer & Phaswana-Mafuya^(52)QN^ *	Mixed adult
			Energy intake	Hattingh *et al.* ^(39)^ * ^,(40)^ * ^,(41)QN^ *	Female adult
			Dietary intake	Becquey *et al.* ^(31)QN^ †	Mixed adolescent and adult
			Dietary patterns	Zeba *et al.* ^(60)QN^ *	Mixed adult
			Dietary quality	Sodjinou *et al.* ^(54)^/Sodjinou *et al.* ^(55)QN^ *	Mixed adult
	Biological (9)	Morbidity (1)	Dietary diversity	Kiboi *et al.* ^(43)QN^ †	Female adolescent and adult
		Age (11)	Fruit and vegetable intake	Landais^(44)^/Landais *et al.* ^(45)QN^ ‡	Female adult
			Fruit and vegetable intake	Peltzer & Phaswana-Mafuya^(52)QN^ ‡	Mixed adult
			Dietary quality	Soualem *et al.* ^(56)QN^ *	Mixed adolescent
			Dietary diversity	Becquey *et al.* ^(31)QN^ *, Savy *et al.* ^(53)QN^ *, Codjoe *et al.* ^(33)QN^ ‡ and Cisse-Egbuonye *et al.* ^(32)QN^ ‡	Mixed adolescent and adult; Adult women; Mixed adolescent and adult; Female adolescent and adult
			Meal skipping	Onyiriuka *et al.* ^(51)QN^ †	Female adolescent
			Food choice	Onyiriuka *et al.* ^(51)QN^	Female adolescent
			Dietary patterns	Zeba *et al.* ^(53)QN^ *	Mixed adult
			Energy intake	Hattingh *et al.* ^(39)^/Hattingh *et al.* ^(40)^/Hattingh *et al.* ^(41)QN^ *	Female adult
			Fattening practices	Jafri *et al.* ^(42)QN^ *	Adult women
		Parity (2)	Dietary patterns	Zeba *et al.* ^(54)QN^ *	Mixed adult
			Fruit and vegetable intake	Landais^(42)^/Landais *et al.* ^(45)QN^ ‡	Adult women
		Gender (5)	Dietary quality	Soualem *et al.* ^(56)QN^ *	Mixed adolescent
			Dietary diversity	Codjoe *et al.* ^(33)QN^ †	Mixed adolescent and adult
			Dietary intake	Aounallah-Skhiri *et al.* ^(30)QN^ *	Mixed adolescent and adult
			Fast-food intake	Van zyl *et al.* ^(58)QN^ †	Mixed adult
			Fruit and vegetable intake	Peltzer & Phaswana-Mafuya^(52)QN^ ‡	Mixed adult
		Body composition (2)	Dietary intake	Pradeilles^(62)MM^ ‡	Mixed adolescent and adult
			Fruit and vegetable intake	Peltzer & Phaswana-Mafuya^(52)QN^ ‡	Mixed adult
		Pubertal development (1)	Dietary intake	Pradeilles^(62)MM^	Mixed adolescent and adult
		BMI *z*-score (1)	Dietary intake/Snacking	Feeley *et al.* ^(35)QN^ †	Mixed adolescent
		Fat mass (1)	Dietary intake/Snacking	Feeley *et al.* ^(35)QN^ †	Mixed adolescent
		Health (2)	Food intake	Waswa^(59)QN^ *	Female adult
			Fruit and vegetable intake	Peltzer & Phaswana-Mafuya^(52)QN^ *	Mixed adult
	Demographic (9)	Income (individual/household) (6)	Dietary diversity	Codjoe *et al.* ^(33)QN^ * and Kiboi *et al.* ^(43)QN^ †	Female adolescent and adult
			Dietary intake	Legwegoh *et al.* ^(23)^/Legwegoh *et al.* ^(24)QL^ and Steyn *et al.* ^(57)QN^ †	Mixed adult; Mixed adolescent and adult
			Dietary patterns	Zeba *et al.* ^(54)QN^ ‡	Mixed adult
			Dietary quality	Soualem *et al.* ^(56)QN^ †	Mixed adolescent
		Socio-economic status (individual/household) (13)	Dietary diversity	Becquey *et al.* ^(31)QN^ † and Savy *et al.* ^(53)QN^ *	Mixed adolescent and adult; Female adult
			Dietary intake	Aounallah-Skhiri *et al.* ^(30)QN^ †, Legwegoh *et al.* ^(23)^/Legwegoh *et al.* ^(24)QL^, Hattingh *et al.* ^(39)^/Hattingh *et al.* ^(40)^/Hattingh *et al.* ^(41)QN^ ‡, Mbochi *et al.* ^(48)QN^ †, Njelekela *et al.* ^(50)QN^ ‡, Pradeilles^(62)MM^ ‡ and Steyn *et al.* ^(57)QN^ †	Mixed adolescent and adult; Mixed adult; Female adult; Female adult; Mixed adult; Mixed adolescent and adult; Mixed adolescent and adult;
			Fruit and vegetable intake	Landais^(44)^/Landais *et al.* ^(45)QN^ *	Female adult
			Dietary quality	Fokeena & Jeewon^(36)QN^ *	Mixed adolescent
			Meal skipping /Food choices	Onyiriuka *et al.* ^(51)QN^ *	Female adolescent and adult
			Fast-food intake	Van zyl *et al.* ^(58)QN^ †	Mixed adult
		Employment (individual/parent/household head) (7)	Dietary diversity	Kiboi *et al.* ^(43)QN^ †, Cisse-Egbuonye *et al.* ^(32)QN^ † and Codjoe *et al.* ^(33)QN^ *	Female adolescent and adult; Female adolescent and adult; Mixed adult and adolescent
			Fruit and vegetable intake	Landais^(44)^/Landais *et al.* ^(45)QN^ †	Female adult
			Dietary intake	Aounallah-Skhiri *et al.* ^(30)QN^ † and Steyn *et al.* ^(57)QN^ †	Mixed adolescent and adult; Mixed adolescent and adult
			Dietary quality	Soualem *et al.* ^(56)QN^ †	Mixed adolescent
		Education (individual/parent) (9)	Dietary diversity	Kiboi *et al.* ^(43)QN^ †	Female adolescent and adult
			Dietary intake	Aounallah-Skhiri *et al.* ^(30)QN^ †, Glozah & Pevalin^(37)QN^ † and Lopez *et al.* ^(46)QN^ *	Mixed adolescent and adult; Mixed adolescent and adult; Mixed adolescent and adult
			Dietary quality	Soualem *et al.* ^(56)QN^ ‡	Mixed adolescent
			Dietary patterns	Zeba *et al.* ^(54)QN^ ‡	Mixed adult
			Fruit and vegetable intake	Landais^(44)^/Landais *et al.* ^(45)QN^ and Peltzer & Phaswana-Mafuya^(52)QN^ †	Female adult ; Mixed adult
			Household dietary diversity	Codjoe *et al.* ^(33)QN^ †	Mixed adolescent and adult
		Wealth (individual/household) (3)	Fruit and vegetable intake	Peltzer & Phaswana-Mafuya^(52)QN^ *	Mixed adult
			Dietary diversity	Codjoe *et al.* ^(33)QN^ †	Mixed adult and adolescent
			Food choice	Agbozo *et al.* ^(28)QN^ *	Mixed adult
		Land ownership (1)	Dietary diversity	Kiboi *et al.* ^(43)QN^ †	Female adolescent and adult
		Ethnicity (5)	Dietary intake	Steyn *et al.* ^(57)QN^ ‡	Mixed adolescent and adult
			Disordered eating	Gitau *et al.* ^(38)QN^ ‡	Male adolescent
			Meal skipping/Food choice	Onyiriuka *et al.* ^(51)QN^ *	Female adolescent and adult
			Fruit and vegetable consumption	Peltzer & Phaswana-Mafuya^(52)QN^ †	Mixed adult
			Dietary diversity	Codjoe *et al.* ^(33)QN^ ‡	Mixed adult and adolescent
		Household food expenditure (2)	Dietary diversity	Becquey *et al.* ^(31)QN^ † and Codjoe *et al.* ^(33)QN^ ‡	Mixed adolescent and adult; Mixed adult and adolescent
		Financial insecurity (1)	Unhealthy eating choice	Draper *et al.* ^(22)QL^	Female adult
Social environment (11)	Family (9)	Marital status (6)	Fruit and vegetable intake and diversity	Landais^(44)^/Landais *et al.* ^(45)QN^ ‡ and Peltzer & Phaswana-Mafuya^(52)QN^ *	Female adult; Mixed adult
			Fattening practices	Rguibi & Behalsen^(25)QL^ and Jafri *et al.* ^(42)QN^ *	Female adolescent and adult; Adult women
			Dietary diversity	Becquey *et al.* ^(31)QN^ † and Savy *et al.* ^(53)QN^ *	Mixed adolescent and adult; Female adult
		Household social roles (1)	Snacking	Batnitzky^(18)QL^	Mixed adult
		Household composition (4)	Meal skipping	Onyiriuka *et al.* ^(51)QN^ *	Female adolescent and adult
			Food intake	Batnitzky^(18)QL^	Mixed adult
			Dietary diversity	Codjoe *et al.* ^(33)QN^ ‡ and Cisse-Egbuonye *et al.* ^(32)QN^ ‡	Mixed adult and adolescent; Female adolescent and adult
		Eating companions (2)	Meal skipping	Onyiriuka *et al.* ^(51)QN^ *	Female adolescent and adult
			Food choice	Brown *et al.* ^(20)QL^	Mixed adolescent and adult
		Shared bowl (1)	Fruit and vegetable intake and diversity	Landais^(43)^/Landais *et al.* ^(45)QN^ ‡	Female adult
		What rest of family eat (2)	Food choice	Charlton *et al.* ^(61)MM^ and Boatemma *et al.* ^(19)QL^	Female adolescent and adult; Mixed adolescent and adult
		Number of children (1)	Fruit and vegetable intake and diversity	Landais^(44)^/Landais *et al.* ^(45)QN^ *	Female adult
		Parental influence (1)	Adequacy of food intake	Waswa^(59)QN^ *	Female adult
		Support in the household (3)	Food choice	Boatemma *et al.* ^(19)QL^, Becquey *et al.* ^(31)QN^ † and Savy *et al.* ^(53)QN^ *	Mixed adolescent and adult; Mixed adolescent and adult; Female adult
			Dietary intake	Glozah & Pevalin^(37)QN^ †	Mixed adolescent and adult
	Friends and peers (*n* 2)	Friendship (4)	Fruit and vegetable consumption	Peltzer & Phaswana-Mafuya^(52)QN^ *	Mixed adult
			Dietary intakes	Sedibe *et al.* ^(26)^ */Voorend *et al.* ^(27)QL^ *	Female adolescent
			Food choice	Boatemma *et al.* ^(19)QL^	Mixed adolescent and adult
			Adequacy of food intake	Waswa^(59)QN^ *	Female adult
			Fast-food intake	Van zyl *et al.* ^(58)QN^ *	Mixed adult
		Religious groups (1)	Dietary intake	Pradeilles^(62)MM^	Mixed adolescent and adult
Physical environment(12)	Home (4)	Household food stocks (1)	Dietary diversity	Becquey *et al.* ^(31)QN^ †, Kiboi *et al.* ^(43)QN^ † and Codjoe *et al.* ^(33)QN^ ‡	Mixed adolescent and adult; Female adolescent and adult; Mixed adult and adolescent
		Food availability (3)	Adequacy of food intake	Waswa^(59)QN^ *	Female adult
			Dietary diversity	Codjoe *et al.* ^(33)QN^ †	Mixed adult and adolescent
			Food choice	Agbozo *et al.* ^(28)QN^ *	Mixed adult
		Living area (3)	Fruit and vegetable intake/ diversity	Landais^(44)^/Landais *et al.* ^(45)QN^ *	Female adult
			Fruit and vegetable intake	Peltzer & Phaswana-Mafuya^(52)QN^ *	Mixed adult
			Food choice	Mayen *et al.* ^(47)QN^ †	Mixed adult
		Housing conditions (2)	Dietary intake	Steyn *et al.* ^(57)QN^ †	Mixed adolescent and adult
			Meal skipping	Onyiriuka *et al.* ^(51)QN^ *	Female adolescent and adult
	Neighbourhoods (7)	Household sanitation (1)	Dietary diversity	Becquey *et al.* ^(31)QN^ † and Savy *et al.* ^(53)QN^ *	Mixed adolescent and adult; Female adult
		Neighbourhood SES (2)	Dietary intake	Pradeilles^(62)MM^ ‡	Mixed adolescent and adult
			Dietary intake/Snacking	Feeley *et al.* ^(35)QN^ *	Mixed adolescent
		Affordability (2)	Food choice	Boatemma *et al.* ^(19)QL^ and Sedibe *et al.* ^(26)^/Voorend *et al.* ^(27)QL^	Mixed adolescent and adult; Female adolescent
		Eating outside of home (2)	Fruit and vegetable consumption	Landais^(43)^/Landais *et al.* ^(45)QN^ *	Female adult
			Dietary diversity	Codjoe *et al.* ^(33)QN^ †	Mixed adult and adolescent
		Where food is bought (1)	Dietary intake	Steyn *et al.* ^(57)QN^ †	Mixed adolescent and adult
		Convenience (2)	Dietary intake	Sedibe *et al.* ^(26)QL^/Voorend *et al.* ^(27)QL^	Female adolescent
			Fast-food intake	Van Zyl *et al.* ^(58)QN^ *	Mixed adult
		Availability (3)	Fast-food intake	Van Zyl *et al.* ^(58)QN^ *	Mixed adult
			Fruit and vegetable intake	Peltzer *et al.*^(52)QN^*	
			Food choices	Boatemma *et al.* ^(19)QL^	Mixed adolescent and adult
	School (2)	School attendance (1)	Dietary habits	Sedibe *et al.* ^(26)^/Voorend *et al.* ^(27)^	Female adolescent
			Dietary intake	Aounallah-Skhiri *et al.* ^(30)QN^ †	Mixed adolescent and adult
Macro-level environment(9)	Food marketing and media (3)	Advertising (1)	Dietary intake	Legwegoh *et al.* ^(23)^/Legwegoh *et al.* ^(23)QL^	Mixed adults
		Media (3)	Fast-food intake	Van Zyl *et al.* ^(58)QN^ *	Mixed adult
			Dietary intake/Disordered eating	Amenyah & Michels^(29)QN^ *	Mixed adolescent
			Food intake	Waswa^(59)QN^ *	Female adult
		Ideal body size (2)	Dietary intake/Disordered eating	Amenyah & Michels^(29)QN^ *	Mixed adolescent
			Disordered eating	Gitau *et al.* ^(38)QN^ ‡	Male adolescent
	Societal and cultural norms/values (2)	Religion (5)	Fruit and vegetable intake	Peltzer *et al.* ^(52)QN^ ‡	Mixed adult
			Skipping meal	Mogre *et al.* ^(49)QN^ *	Mixed adult
			Dietary diversity	Becquey *et al.* ^(31)QN^ †, Savy *et al.* ^(53)QN^ * and Codjoe *et al.* ^(33)QN^ ‡	Mixed adolescent and adult; Female adult; Mixed adult and adolescent
			Food intake	Waswa^(59)QN^ *	Female adult
		Cultural beliefs (4)	Food intake	Waswa^(59)QN^ *	Female adult
			Fattening practices	Rguibi & Behalsen^(25)QL^	Female adolescent and adult
			Dietary diversity	Codjoe *et al.* ^(33)QN^ ‡	Mixed adult and adolescent
			Dietary intake	Legwegoh *et al.* ^(23)^/Legwegoh *et al.* ^(23)QL^	Mixed adults
	Food and beverage industry (4)	Food prices (5)	Dietary intake	Legwegoh *et al.* ^(23)^/Legwegoh *et al.* ^(23)QL^ and Sedibe *et al.* ^(26)^/Voorend *et al.* ^(27)QL^	Mixed adults; Female adolescent
			Food choice	Charlton *et al.* ^(61)MM^	Female adolescent and adult
			Food intake	Waswa^(59)QN^ *	Female adult
			Unhealthy eating choice	Draper *et al.* ^(22)QL^	Female adult
		Quality/freshness of food (1)	Food choice	Charlton *et al.* ^(61)QN^ *	Female adolescent and adult
		Quick/easy to make foods (1)	Food choice	Charlton *et al.* ^(61)MM^	Female adolescent and adult
		Presentation and packaging (1)	Food choice	Charlton *et al.* ^(61)MM^	Female adolescent and adult

MM, mixed methods; QN, quantitative study; QL, qualitative study.

* Association not assessed/reported.

† Significant association.

‡ Association assessed but NS.

### Dietary factors in adult women, adult men and adolescents

#### Individual level

**Fig. 2:**
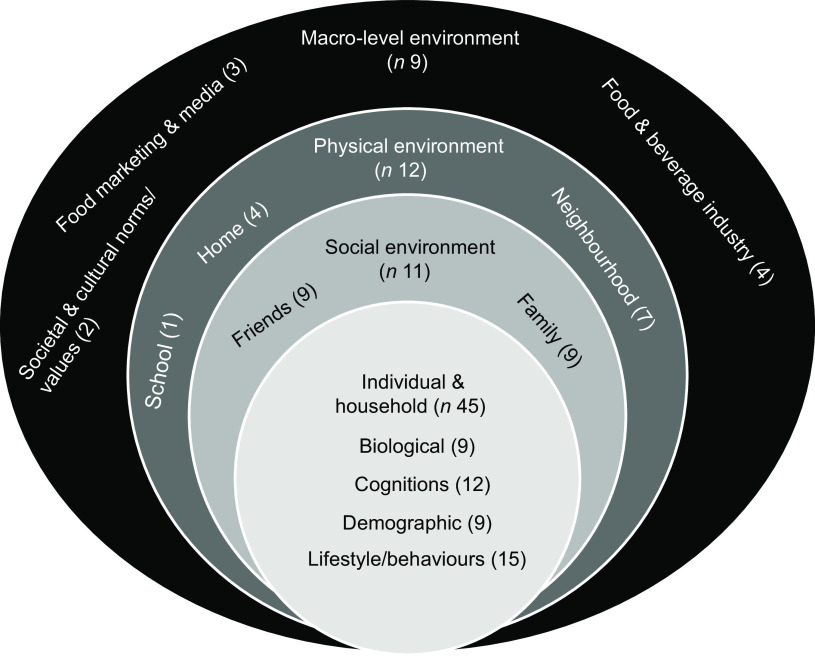
A summary of factors (*n* 77) emerging from the included studies at different environmental levels

Almost two-thirds of factors identified were on the individual level (45/77), of which twelve related to cognitions, fifteen to lifestyle/behaviours, nine were biological factors and nine were demographic factors (Fig. 2). Factors specific to adolescents included self-esteem, body satisfaction, dieting, spoken language, school attendance, gender, body composition, pubertal development, BMI and fat mass.

##### Cognitions

Taste and hunger were cognition-related factors only found within adult studies^(26,27,32,58,61)^. For instance, one quantitative study^(58)^ in Johannesburg found that 52·5 % of participants believed taste influenced fast-food intake. Higher perceived stress levels were found to significantly decrease the amount of fruit and vegetable consumption in a mixed adult population in Egypt, with a more pronouned effect in men^(34)^. Food knowledge and subjective health status was more commonly reported in the studies of adults^(28,46,59)^. Preferences, mood and perception of diet quality and quantity were reported in both qualitative and quantitative studies of both adolescents and adults^(19,26,27,31,59)^.

A small number of factors emerged on the relationship between body satisfaction and dietary behaviours. An association was identified between decreased self-esteem and body satisfaction with disordered eating in South African adolescents, as measured by the Eating Attitudes Tests 26^(38)^. No significant association was found between body image perception and food intake in a quantitative study of female adults^(59)^.

##### Lifestyle/behaviours

A third of individual-level factors identified for adults were categorised under the lifestyle/behaviours sub-level. Time limitation was found to be an important factor in five studies encompassing qualitative and quantitative data conducted in Botswana, Cape Verde, Ghana and South Africa^(20,21,23,24,49,58)^. In the qualitative study conducted in Cape Verde^(^ ^21)^, reduced time availability was associated with the intake of unhealthy street foods. Other important lifestyle-related factors identified in a quantitative study related to lack of fruit and vegetable intake^(52)^ were tobacco use, alcohol use, physical inactivity and low quality of life. Spoken language was found to be significantly associated with dietary quality in one quantitative study conducted in Morocco, as adolescents who only spoke Arabic had a poorer quality of diet than those who spoke both Arabic and French^(56)^.

##### Biological

Evidence from quantitative studies was found for the role of biological factors, which were associated with dietary behaviours in adults, that is, morbidity^(43)^, age^(31,39–42,44,45,51,53,56)^ and having multiple children (parity)^(44,45,54)^. For instance, increased morbidity was significantly associated with minimum dietary diversity among pregnant women in Kenya^(43)^.

More diverse biological factors were investigated for adolescents than for adults. However, only age^(51)^, BMI and fat mass^(35)^ were significantly associated with dietary behaviours. For instance, increasing age was significantly associated with skipping meals among schoolgirls in Nigeria^(51)^ and fat mass was negatively associated with poor eating behaviour^(35)^.

##### Demographic

More demographic factors were identified in adult women than in mixed adult studies. In one quantitative study of adults conducted in Burkina Faso, males of higher SES, as measured by income and education were significantly aggregated in the ‘urban’ diet cluster, while there were proportionally more lower income, non-educated and female subjects in the ‘traditional’ diet cluster^(54)^. Other factors that were investigated were household composition and family profession, but their relationship with dietary behaviours was NS. Adolescents with high SES adhered to more aspects of dietary guidelines than those of low SES in one quantitative study in Mauritius^(36)^.

Qualitative and quantitative studies have found that the importance of household SES was apparent across a range of SES indicators including household income or wealth^(23,24,33,43,50,54,57)^, employment^(32,43,45,56,57)^, land ownership^(43)^ and financial insecurity^(22)^. Educational level of individuals or parents was also found to play a role in dietary behaviours in several quantitative studies^(30,33,37,43–46,52,54,56)^. Higher parental education level was associated with better dietary intake in four quantitative studies among adolescents^(30,33,37,46)^, resulting in a higher modern dietary diversity score for adolescents in Tunisia,^(30)^ higher household dietary diversity score in Ghana^(33)^ and better healthy eating behaviours in Ghana^(37)^ and Morocco^(46)^ than those whose parents had average or low educational attainment.

Dietary behaviours were associated with ethnicity in South African adults^(38,52)^ and adolescents in South Africa^(38)^ and Nigeria^(51)^.

#### Social environment

Eleven factors emerged that related to the social environment, eleven studies (both qualitative and quantitative) explored family influences^(18–20,25,31,42,44,45,51,53,59,61)^ and four studies investigated friendship^(19,26,27,52,59)^ (Fig. 2).

##### Family

The social environment was particularly investigated in adolescent studies; nine factors related to the family including marital status, with evidence coming from both qualitative and quantitative studies^(25,31,42,44,53)^, what the rest of the family eats^(19,61)^ and support in the household^(19,31,53)^.

##### Friends

Two qualitative studies examined the role of friendship on dietary habits and reported that friendship was associated with dietary habits in South African adolescents^(26,27)^, stating that ‘participants often ate the same food as their friends’ and that shared food consumption between friends was common. In another qualitative study in Ghana, some participants mentioned friends as influencing food choice; foods recommended among peers were usually processed foods such as savoury snacks, soda and instant noodles^(19)^. A quantitative study conducted among South African adults^(52)^ did not find a significant association between social cohesion and fruit and vegetable consumption.

#### Physical environment

Fourteen studies (qualitative and quantitative) investigated the role of the physical environment on dietary behaviours, of which nine included adolescents^(19,26,27,31,33,35,43,51,57,62)^. Twelve factors emerged in the physical food environment that influenced dietary behaviours. Seven of these were in the neighbourhood, four in the home environment and one in the school environment (Fig. 2).

Convenience and availability of food were the most investigated factors in the physical environment. For instance, convenience was identified as a factor influencing fast-food intake with one quantitative study in South Africa noting that 58·1 % of participants believed it influenced their food choices^(58)^. Significant associations were found between housing conditions and where food is bought with dietary behaviours in South Africa^(57)^. Two studies found an association between eating outside the home and dietary behaviours^(33,44,45)^. Eating outside the home was associated with higher household dietary diversity in a quantitative study in Ghana, while food eaten at home was associated with lower household dietary diversity scores^(33)^.

The influence of school on dietary habits was investigated by only one qualitative study^(26)^, which found that availability of food within schools, as well as sharing food within school, influenced dietary habits in South Africa.

#### Macro-environment

Nine factors emerged as influencing dietary behaviours that were on the macro-environment level. Three of these factors related to the food marketing and media environment, two related to societal and cultural values and four related to the role of the food and beverage industry.

Food prices were associated with fast-food intake in one South African quantitative study of young adults^(58)^. Media and advertising were found to be associated with dietary intake of adults in both qualitative and quantitative studies in Botswana^(23,24)^ and South Africa^(58)^. About 49 % of participants in one study in South Africa stated that they believed media messages influenced their decision to purchase fast food^(58)^. In a quantitative study conducted in South Africa, ideal body size was related to dietary behaviours^(38)^. A quantitative study conducted in Ghana^(29)^ identified that larger ideal body size was associated with a changed Eating Attitudes Tests 26 score. Lack of religious involvement was associated with dietary behaviour in one quantitative study of adults in South Africa^(52)^, and one quantitative study of adults and adolescents in Ghana but was not associated with meal skipping or food choices in adults^(49)^.

## Discussion

This systematic mapping review mapped the factors influencing dietary behaviours of adolescents and adults in African urban food environments and identified areas for future research. Thirty-nine studies (forty-five records) were included in the final data synthesis. In total, seventy-seven factors influencing dietary behaviours were identified, with two-thirds at the individual level (45/77). Factors in the social (11/77), physical (12/77) and macro (9/77) environments were investigated less. The inclusion of two additional population groups (adult men and adolescents), in comparison to the original review, expands the generalisability of findings to the general population in urban Africa. Studies included in this review were from fifteen African countries, encompassing a range of low-, middle- and high-income African countries, reflecting the heterogeneity of urban African contexts. However, over half (22/39) were conducted in Ghana, Morocco or South Africa. This updates and extends a previous review, which was restricted to women^(8)^. The current review updated and extended the demographic scope to include men and adolescents, as well as women.

Findings synthesised from included studies indicate that the most investigated factors for adults and adolescents were the individual and household environment of the socio-ecological model as described by Story *et al.* ^(15)^. This finding is consistent with our previous review^(8)^. Dietary behaviour was significantly associated with a range of individual and household environmental factors: household income, educational level, employment, land ownership, socio-economic status (SES), ethnicity and financial insecurity. Low self-esteem, high levels of stress and lack of time were associated with unhealthy dietary behaviours. The focus on individual-level factors might be attributable to the fact that promoting healthy eating and preventing obesity have predominantly focused on changing behaviour through interventions such as nutrition education, although such interventions alone have met with little success^(63)^.

Studies involving adolescents investigated factors in their social environments and were less focused on the role of the physical food environment on dietary behaviours, than for adults. This bias is unsurprising given that adolescence is defined as a transient formative period where many life patterns are learnt^(64)^, particularly through the social environment. Shared food consumption between adolescent friends was common. Evidence from the wider literature outlines the social transmission of eating behaviours, whereby a strong relationship exists between the social environment and amount or types of food eaten^(65)^. This implies individuals tend to eat according to the usual social group they find themselves, either in terms of quantity or types of food eaten^(66)^. Thus, understanding the role of the social environment among adults and adolescents as a modifiable factor influencing dietary behaviours offers an opportunity for developing nutrition interventions that harness social relationships.

Convenience and availability of food were the most investigated factors in the physical environment. Significant associations were found between housing conditions and dietary intake, and where food was purchased and dietary intake. In contrast to the socio-ecological model^(15)^, our map lacks evidence for the role of several factors in the physical environment such as workplaces, schools (one study), supermarkets and convenience stores.

In contrast to studies conducted in high-income countries, factors influencing dietary behaviours in the macro-environment were rarely investigated in our review for adults or adolescents. Only food/drink advertising and religion (adolescents only) and food prices were associated with unhealthy dietary behaviours, but many macro-level factors are known to influence diet, such as the political context, economic systems, health care systems and behavioural regulations^(67)^ that were not studied. One possible explanation may be that because Story’s model was generated following research within high-income counties, some of the sub-levels may be less relevant to the African context. Factors that have been shown to influence dietary behaviours in high-income countries and were investigated in studies included in this review include food prices, social networks (friendship), time constraints and convenience. However, in high-income countries these factors are often reported in low-income groups^(68)^. Another important finding from this review is the consistent association between SES and dietary behaviours as expected. SES is a global concern, and several studies have shown that lower SES restricts food choices, thus compelling the consumption of unhealthy foods^(69–71)^.

Of the thirty-nine studies identified, none specifically investigated adult men, as they were only included in mixed adult population studies. Adult men and women studies identified during this review showed similar types of factors associated with dietary behaviour across the different environments, suggesting that similar interventions could be targeted at both men and women. However, demographic factors were identified more in adult women than in mixed adult studies. This implies that the household is an important setting in which to reach women. The findings for women from this review went beyond that of the previous review. Three more factors (stress, self-esteem and body satisfaction) were identified in the updated review. Furthermore, the expanded review identified evidence of more physical-level dietary factors including housing, living area, convenience and where food is bought.

As the most common study methodology of included studies was cross-sectional, it is not possible to conclude on causality of the factors in different components of the food environment on dietary behaviours. Limitations regarding the use of the socio-ecological model^(68)^ became evident during the review, as there is overlap between the different environmental levels for factors such as SES, spoken language and religious group. For instance, SES crosses multiple levels of the model, particularly in adolescents, as SES is often measured via physical or household/family-related factors. Another example is religious groups, which do not fit within the current sub-categories defined by Story’s ecological model^(15)^. Although religion may broadly be classified as a factor in the macro-environment, religious groups may best fit in the social environment. While the socio-ecological model depicts reality as artificially separating individual and social experiences^(68)^, it is still a useful tool to communicate with policy makers and practitioners, unlike systems-based approaches, which are better at representing reality but rely on data on causality and mechanisms that are often lacking in cross-sectional and quantitative studies^(72)^ and are harder to communicate to a non-expert audience.

This review revealed considerable heterogeneity in the design of quantitative studies and the outcome measures used for assessing dietary behaviours. Future quantitative studies should ensure that outcome measures are clearly defined and report the direction of association between the factors examined and whether dietary behaviours are healthy or unhealthy. Quantitative studies should enhance the control of confounding variables to prevent them from introducing bias into the findings, and longitudinal quantitative studies are needed to be able to measure how factors influencing dietary behaviours are changing with the transformation of food environments. Qualitative studies are useful for understanding the complex relationships between determinants of dietary behaviours. Qualitative studies need to have a rigorous design and improve the reporting of reflexivity by considering the impact of the role of researcher characteristics on the data collected to improve their quality.

This review highlights the need for robust mixed methods studies to gain a better understanding of the drivers of dietary behaviours in urban food environments in Africa.

This is the first systematic mapping review that focuses on environmental factors of dietary behaviour for all population groups in an urban African context. The nutrition transition has been associated with changes in dietary patterns globally with concomitant increases in obesity and NR-NCD, now among the leading causes of death^(73)^. In African countries, NR-NCD risk is increasing at a faster rate and at a lower economic threshold than seen in high-income countries^(74)^, hence the need for this review that identifies context specific factors that influence dietary behaviours. The recent focus on good health and well-being as part of the Sustainable Development Goals (SDG3)^(^ ^75)^ also reflects this review’s aim to identify the underlying determinants of dietary behaviour in the urban African context to identify avenues for interventions.

## Conclusion

The relatively small number of appropriate studies identified, following an extensive literature search, indicates a significant gap in research into understanding of the factors influencing diets in food environments in urban Africa. Due to the increasing presence of multiple burdens of malnutrition in urban Africa, secondary to the nutrition transition^(6)^, more studies should be directed at investigating how food environments are changing and driving this complex nutritional landscape. In particular, future research could emphasise the investigation of adult men and adolescents. The evidence from this review will contribute towards developing a socio-ecological framework of factors influencing dietary behaviours adapted to urban African food environments.

## Supplementary material

For supplementary material accompanying this paper visit https://doi.org/10.1017/S1368980019005305. click here to view supplementary material
