# Supplementary material for: Factors influencing dietary behaviours in urban food environments in Africa: a systematic mapping review
Source: Public Health Nutr. 2020 May 26;23(14):2584–601. doi: 10.1017/S1368980019005305 (PMC7116038; doi:10.1017/S1368980019005305)
Supplement: Supplementary file 1 [file S1368980019005305sup.zip › S1368980019005305sup001.docx]

Additional Table 2: Quality assessment scores for qualitative studies. Yes- 2, Partial- 1, No- 0, Not applicable-NA

|  | **Question/ objective** | **Study design** | **Context** | **Theoretical framework** | **Sampling** | **Data collection** | **Data analysis** | **Verification procedure** | **Conclusions** | **Reflexivity** |
| --- | --- | --- | --- | --- | --- | --- | --- | --- | --- | --- |
| Batnitzky *et al.* (2008)^(16)^ | **2** | **2** | **2** | **2** | **1** | **1** | **0** | **0** | **2** | **0** |
| Boatemaa *et al.* (2018)^(17)^ | **2** | **2** | **2** | **2** | **1** | **2** | **2** | **0** | **2** | **0** |
| Brown *et al.* (2015)^(18)^ | **2** | **2** | **2** | **2** | **2** | **2** | **2** | **0** | **2** | **0** |
| *Charlton *et al.* (2014)^(59)^ | **2** | **1** | **1** | **2** | **1** | **2** | **1** | **0** | **2** | **0** |
| Craveriro *et al.* (2016)^(19)^ | **1** | **2** | **2** | **1** | **2** | **2** | **2** | **2** | **2** | **0** |
| Draper *et al.* (2016)^(20)^ | **1** | **2** | **2** | **2** | **2** | **2** | **2** | **2** | **2** | **0** |
| Legwegoh *et al.* (2012)^(22)^, (2016)^(22)^ | **1** | **2** | **2** | **2** | **2** | **2** | **1** | **0** | **1** | **0** |
| *Pradeilles (2015) ^(60)^ | **2** | **2** | **2** | **2** | **2** | **2** | **2** | **2** | **2** | **0** |
| Rguibi and Behalsen, 2006^(23)^ | 2 | 2 | 1 | 2 | 1 | 1 | 0 | 0 | 2 | 0 |
| Sedibe *et al.* (2014)^(24)^; Voorend *et al.* (2013)^(25)^ | **2** | **2** | **2** | **2** | **2** | **2** | **2** | **2** | **2** | **0** |
| ***mixed methods study- scored here for qualitative component. Quality appraisal was conducted using**  **using a validated quality assessment tool^(13).^** | | | | | | | | | | |
