# Supplementary material for: Factors influencing dietary behaviours in urban food environments in Africa: a systematic mapping review
Source: Public Health Nutr. 2020 May 26;23(14):2584–601. doi: 10.1017/S1368980019005305 (PMC7116038; doi:10.1017/S1368980019005305)
Supplement: Supplementary file 1 [file S1368980019005305sup.zip › S1368980019005305sup002.docx]

Additional Table 3: Quality assessment scores for quantitative studies. Yes- 2, Partial- 1, No- 0, Not applicable-NA.

|  | **Question/ objective** | **Study design** | **Subject selection** | **Subject characteristics** | **Random allocation** | **Investigator blinding** | **Subject blinding** | **Outcome measure** | **Sample size** | **Data analysis** | **Estimate of variance** | **Control for confounding** | **Result reporting** | **Conclusions** |
| --- | --- | --- | --- | --- | --- | --- | --- | --- | --- | --- | --- | --- | --- | --- |
| Agbozo *et al.* (2018)^(26)^ | 2 | 2 | 2 | 2 | NA | NA | NA | 2 | 2 | 2 | 0 | 0 | 2 | 1 |
| Amenyah *et al.* (2016)^(27)^ | 2 | 2 | 2 | 2 | NA | NA | NA | 2 | 2 | 2 | 2 | 1 | 2 | 2 |
| Aounalla-Sikhiri *et al.* (2011)^(28)^ | 2 | 2 | 2 | 2 | NA | NA | NA | 2 | 2 | 2 | 1 | 2 | 2 | 1 |
| Becquey *et al.* (2010)^(29)^ | 2 | 2 | 2 | 1 | NA | NA | NA | 2 | 2 | 2 | 1 | 2 | 2 | 2 |
| *Charlton *et al.* (2004)^(59)^ | 2 | 2 | 1 | 2 | N/A | N/A | N/A | 1 | N/A | 0 | N/A | N/A | 2 | 2 |
| Cisse-Egbuonye *et al.* (2017)^(30)^ | 2 | 2 | 2 | 2 | NA | NA | NA | 1 | 2 | 2 | 2 | 2 | 2 | 1 |
| Codjoe *et al.* (2016)^(31)^ | 2 | 2 | 1 | 2 | NA | NA | NA | 2 | 2 | 2 | 2 | 2 | 2 | 1 |
| El Ansari *et al.* (2015)^(32)^ | 2 | 2 | 1 | 2 | NA | NA | NA | 2 | 2 | 2 | 2 | 2 | 2 | 2 |
| Feeley *et al.* (2013)^(33)^ | 1 | 2 | 1 | 2 | NA | NA | NA | 1 | 2 | 2 | 1 | 2 | 2 | 2 |
| Fokeena *et al.* (2012)^(34)^ | 2 | 2 | 1 | 1 | NA | NA | NA | 2 | 1 | 2 | 2 | 0 | 2 | 2 |
| Glozah *et al.* (2015)^(35)^ | 2 | 1 | 1 | 2 | NA | NA | NA | 1 | 2 | 2 | 2 | 2 | 2 | 1 |
| Gitau *et al.* (2014)^(36)^ | 2 | 2 | 2 | 1 | NA | NA | NA | 2 | 1 | 2 | 0 | 0 | 2 | 1 |
| Hattingh *et al.* 2006^(37)^; 2011^(38)^ ; 2014^(39)^ | 1 | 2 | 2 | 2 | N/A | N/A | N/A | 1 | 1 | 2 | 1 | N/A | 2 | 2 |
| Jafri *et al.* (2013)^(40)^ | 2 | 2 | 2 | 0 | N/A | N/A | N/A | 1 | 1 | 2 | 0 | 0 | 2 | 2 |
| Kiboi *et al.* (2017)^(41)^ | 1 | 2 | 2 | 2 | NA | NA | NA | 2 | 2 | 2 | 2 | 2 | 2 | 2 |
| Landais *et al.* 2012^(42)^;(2015)^(43)^ | 2 | 2 | 2 | 2 | NA | NA | NA | 2 | 2 | 2 | 2 | 1 | 2 | 1 |
| Lopez *et al.* (2012)^(44)^ | 2 | 2 | 1 | 2 | NA | NA | NA | 2 | 2 | 2 | 0 | 0 | 2 | 0 |
| Mayen *et al.* (2016)^(45)^ | 1 | 2 | 2 | 2 | NA | NA | NA | 1 | 2 | 2 | 1 | 0 | 2 | 0 |
| Mbochi *et al.* (2012)^(46)^ | 2 | 1 | 2 | 1 | N/A | N/A | N/A | 1 | 2 | 2 | 1 | 0 | 1 | 2 |
| Mogre *et al.* (2013)^(47)^ | 2 | 2 | 2 | 2 | N/A | N/A | N/A | 1 | 1 | 2 | 0 | N/A | 2 | 2 |
| Njelekela *et al.* (2011)^(48)^ | 1 | 2 | 2 | 1 | NA | NA | NA | 2 | 2 | 2 | 1 | 0 | 2 | 1 |
| Onyririuka *et al*. (2013)^(49)^ | 2 | 2 | 2 | 2 | NA | NA | NA | 2 | 2 | 2 | 2 | 0 | 2 | 2 |
| Peltzer *et al.* (2012)^(50)^ | 2 | 2 | 1 | 2 | NA | NA | NA | 2 | 2 | 2 | 0 | 0 | 2 | 2 |
| *Pradeilles (2015)^(60)^ | 2 | 2 | 2 | 2 | NA | NA | NA | 2 | 2 | 2 | 2 | 2 | 2 | 2 |
| Savy *et al.* (2008)^(51)^ | 2 | 2 | 2 | 2 | N/A | N/A | N/A | 2 | 1 | 2 | 2 | 2 | 2 | 2 |
| Sodjinou *et al.* 2008^(52)^; 2009^(53)^ | 2 | 2 | 2 | 2 | N/A | N/A | N/A | 2 | 2 | 2 | 2 | 2 | 2 | 2 |
| Soualem *et al.* (2012)^(54)^ | 2 | 2 | 2 | 2 | NA | NA | NA | 2 | 2 | 2 | 2 | 0 | 2 | 1 |
| Steyn *et al.* (2011)^(55)^ | 2 | 2 | 2 | 1 | NA | NA | NA | 2 | 2 | 2 | 2 | 2 | 2 | 2 |
| Van Zyl *et al.* (2010)^(56)^ | 2 | 1 | 1 | 2 | N/A | N/A | N/A | 2 | 2 | 2 | N/A | 0 | 2 | 2 |
| Waswa, 2011^(57)^ | 2 | 2 | 2 | 2 | N/A | N/A | N/A | 1 | 1 | 2 | 2 | 1 | 2 | 2 |
| Zeba et al. (2014)^(58)^ | 2 | 2 | 2 | 2 | NA | NA | NA | 2 | 2 | 2 | 2 | 1 | 2 | 2 |

*****mixed methods study- scored here for quantitative component. Quality appraisal was conducted using a validated quality assessment tool^(13).^
